# Identification of Tissue microRNAs Predictive of Sunitinib Activity in Patients with Metastatic Renal Cell Carcinoma

**DOI:** 10.1371/journal.pone.0086263

**Published:** 2014-01-24

**Authors:** Celia Prior, Jose Luis Perez-Gracia, Jesus Garcia-Donas, Cristina Rodriguez-Antona, Elizabeth Guruceaga, Emilio Esteban, Cristina Suarez, Daniel Castellano, Aránzazu González del Alba, Maria Dolores Lozano, Joan Carles, Miguel Angel Climent, Jose Angel Arranz, Enrique Gallardo, Javier Puente, Joaquim Bellmunt, Alfonso Gurpide, Jose Maria Lopez-Picazo, Alvaro Gonzalez Hernandez, Begoña Mellado, Esther Martínez, Fernando Moreno, Albert Font, Alfonso Calvo

**Affiliations:** 1 Oncology Division, CIMA, University of Navarra, Pamplona, Spain; 2 Oncology Department, Clinica Universidad de Navarra, Pamplona, Spain; 3 Fundacion Hospital de Alcorcon, Madrid, Spain; 4 Human Cancer Genetics Programme, CNIO, Madrid, Spain; 5 Proteomics, Genomics and Bioinformatics Unit, CIMA, University of Navarra, Pamplona, Spain; 6 Medical Oncology Department, Hospital Universitario Central de Asturias, Oviedo, Spain; 7 Medical Oncology Department, Hospital Universitario Vall de Hebron, Barcelona, Spain; 8 Medical Oncology Department, Hospital Universitario 12 de Octubre, Madrid, Spain; 9 Medical Oncology Department, Hospital Universitario Son Espases, Palma de Mallorca, Spain; 10 Pathology Department, University Clinic of Navarra, University of Navarra, Pamplona, Spain; 11 Medical Oncology Department, Instituto Valenciano de Oncología, Valencia, Spain; 12 Medical Oncology Department, Hospital Universitario Gregorio Marañon, Madrid, Spain; 13 Medical Oncology Department, Parc Taulí Sabadell Hospital Universitari, Sabadell, Spain; 14 Medical Oncology Department, Hospital Clinico Universitario San Carlos, Madrid, Spain; 15 Medical Oncology Department, University Hospital del Mar, Barcelona, Spain; 16 Biochemistry Department, Clinica Universidad de Navarra, Pamplona, Spain; 17 Medical Oncology Department, IDIBAPS, Hospital Clinic, Barcelona, Spain; 18 Medical Oncology Department, Hospital de Jaen, Jaen, Spain; 19 Medical Oncology Service, Institut Català d'Oncologia, Hospital Germans Trias i Pujol, Badalona, Spain; The University of Tennessee Health Science Center, United States of America

## Abstract

**Purpose:**

To identify tissue microRNAs predictive of sunitinib activity in patients with metastatic renal-cell-carcinoma (MRCC) and to evaluate *in vitro* their mechanism of action in sunitinib resistance.

**Methods:**

We screened 673 microRNAs using TaqMan Low-density-Arrays (TLDAs) in tumors from MRCC patients with extreme phenotypes of marked efficacy and resistance to sunitinib, selected from an identification cohort (n = 41). The most relevant differentially expressed microRNAs were selected using bioinformatics-based target prediction analysis and quantified by qRT-PCR in tumors from patients presenting similar phenotypes selected from an independent cohort (n = 101). In vitro experiments were conducted to study the role of miR-942 in sunitinib resistance.

**Results:**

TLDAs identified 64 microRNAs differentially expressed in the identification cohort. Seven candidates were quantified by qRT-PCR in the independent series. MiR-942 was the most accurate predictor of sunitinib efficacy (p = 0.0074). High expression of miR-942, miR-628-5p, miR-133a, and miR-484 was significantly associated with decreased time to progression and overall survival. These microRNAs were also overexpressed in the sunitinib resistant cell line Caki-2 in comparison with the sensitive cell line. MiR-942 overexpression in Caki-2 up-regulates MMP-9 and VEGF secretion which, in turn, promote HBMEC endothelial migration and sunitinib resistance.

**Conclusions:**

We identified differentially expressed microRNAs in MRCC patients presenting marked sensitivity or resistance to sunitinib. MiR-942 was the best predictor of efficacy. We describe a novel paracrine mechanism through which high miR-942 levels in MRCC cells up-regulates MMP-9 and VEGF secretion to enhance endothelial migration and sunitinib resistance. Our results support further validation of these miRNA in clinical confirmatory studies.

## Introduction

Several drugs have been developed for metastatic renal-cell carcinoma (MRCC) during recent years, establishing a complex scenario in which the choice of the optimal agent for each patient is cumbersome. Therefore, the development of predictive biomarkers to maximize clinical benefit and to spare unnecessary toxicities and costs remains an unmet challenge.

MicroRNAs (miRNAs) are noncoding single-stranded small RNAs (∼22 nucleotides) that regulate posttranscriptional gene expression. MiRNAs levels are altered in many human diseases including cancer, where they can act either as oncogenes or as tumor-suppressor genes, thus playing a crucial role in tumor initiation, progression and metastasis. MiRNAs are highly stable molecules that can be quantified in tissues and body fluids and are therefore considered a promising platform for the development of cancer biomarkers [1]. Different miRNA signatures have been explored for diagnosis, staging and sub-classification of cancer, as well as for predicting prognosis and treatment efficacy. Several studies have addressed their implication in renal-cell carcinoma and recently their importance as potential predictive serum biomarkers of treatment response has been described [2]

The aim of this study was to identify candidate predictive tumor miRNAs in MRCC patients treated with sunitinib, one of the most widely used drugs in this setting, and to further understand their contribution to the molecular mechanisms of sunitinib resistance. We employed the methodology of extreme phenotype selection, which consists of screening potential biomarkers with high-throughput techniques in the patients that present the most informative clinical features, in order to increase the probability of finding molecular factors potentially linked to such phenotypes. We selected the most significant miRNAs and studied them in an independent cohort of MRCC patients treated with sunitinib, to assess their predictive role in this setting. Results were also investigated in *in vitro* systems using MRCC and endothelial cells, as microenvironmental tumor-endothelial cell interactions are essential for response to sunitinib.

## Materials and Methods

### Patient selection and study design

Two cohorts of MRCC patients treated with sunitinib were selected. The screening cohort was obtained from one institution and included 41 patients. The independent cohort was recruited from 15 institutions and included 101 patients [3]. Characteristics of patients selected from both cohorts are shown in Table 1. All patients had received sunitinib 50 mg daily in a standard four week treatment, followed by two week rest continuous schedule. None had received previous therapy either with antiangiogenics or m-TOR inhibitors. Patients were managed according to standard clinical practice. Response was evaluated every two cycles using RECIST criteria. Time to progression (TTP) and overall survival (OS) were monitored from the start of treatment. The study protocol was approved by the Ethical Review Board of Clinica Universidad de Navarra as the Reference Ethics Committee. All patients signed written informed consent.

**Table 1:** Patient characteristics.

Characteristic	Screening cohort		Independent cohort	
	Sensitive	Resistant	Sensitive	Resistant
	N = 3	N = 3	N = 14	N = 6
	(%)	(%)	(%)	(%)
Sex				
Male	2 (67)	1 (33)	12 (86)	2 (33)
Female	1 (33)	2 (67)	2 (14)	4 (67)
Age				
Median	52	47	59	60
Range	(47–59)	(39–61)	(40–70)	(47–81)
Performance status				
0	3 (100)	2 (67)	6 (43)	2 (33)
1	-	1 (33)	7 (50)	4 (67)
2	-	-	1 (7)	-
Tumor histology				
Clear-cell carcinoma	3 (100)	3 (100)	14 (100)	6 (100)
MSKCC prognostic group				
Good	3 (100)	-	6 (43)	-
Intermediate	-	2 (67)	5 (36)	4 (67)
Poor	-	1 (33)	-	1 (16)
Unavailable	-	-	3 (21)	1 (16)
Number of metastatic sites				
Median	2	4	2	2
Range	(2–3)	(2–5)	(1–4)	(1–3)
Sites of metastasis				
Lymph nodes	2 (67)	3 (100)	6 (43)	3 (50)
Lung	3 (100)	2 (67)	10 (71)	6 (100)
Bone	-	3 (100)	4 (29)	1 (16)
Liver	-	1 (33)	-	1 (16)
Kidney	1 (33)	-	1 (7)	-
Response to sunitinib				
Partial response	3 (100)	-	11 (79)	-
Stable disease	-	-	3 (21)	-
Progression	-	3 (100)	-	6 (100)
Median time to progression (months)	32	2	31	3

We used the methodology of extreme phenotype selection [4], [5], [6], [7]. This methodology has previously been used to identify biomarkers of drug related toxicity [8], [9] and sunitinib efficacy [10]. In each cohort we selected two groups of patients with extreme phenotypes of marked sensitivity or resistance to sunitinib. The criteria to select sensitive patients were TTP greater than 22 months (twice the time reported in the sunitinib phase III registration trial [11]) or a partial response greater than 50%. Patients were categorized as extreme phenotypes of sunitinib resistance when TTP was ≤5 months (half the time reported in the phase III registration trial). To ensure that these patients were truly resistant to sunitinib, performance status ≤1 and absence of comorbidities were required for eligibility.

### Taqman Low density array (TLDA) screening

Tumor samples from patients from the screening cohort presenting extreme phenotypes of sunitinib sensitivity (n = 3) and resistance (n = 3) were assayed with TLDAs to identify potential candidate predictive miRNAs. Tissues were formalin-fixed and paraffin-embedded. Following confirmation of tumor presence by a pathologist, total RNA from four unstained sections (10 µm) was extracted with the RecoverAll™ Total Nucleic Acid Isolation Kit (Ambion). The concentration and quality of total RNA were determined by analysis of 1 µl of the RNA solution in a NanoDrop 1000 Spectrophotometer (Thermo Scientific). An average of 20 µg of RNA was obtained.

350 ng of total RNA from each patient was first reverse-transcribed using a stem-loop RT Megaplex Primer Pool and the Taqman MicroRNA Reverse Transcription Kit (Applied Biosystems). cDNAs were preamplified with the TaqMan Preamp Master Mix kit and Megaplex Preamp Primer Human pools. Expression of 667 human mature miRNAs and six endogenous controls was profiled using TLDA microfluidic cards A and B (Applied Biosystems) on a 7900HT Fast real time system. MammU6 expression was used as endogenous control.

Differentially expressed miRNAs (p<0.05) were selected for further study after bioinformatic analysis of target prediction data bases and thorough analysis of bibliography, to select potential candidates that could be involved in MRCC-related pathways and response to sunitinib. The following databases were used for target prediction: TargetScan release 5·1 [12], PicTac [13], PITA release 6 [14], miranda release sept2008 [15], and microcosm released 5 [16].

### MiRNA quantification in tumor samples by qRT-PCR

Selected miRNAs were quantified by qRT-PCR in tumors from patients with extreme phenotypes of sunitinib sensitivity (n = 14) or resistance (n = 6) from the independent series. 20 ng of total RNA and miRNA-specific primers for the selected miRNAs and for RNU6B (endogenous control) were used for stem-loop RT, followed by q-PCR amplification for 40 cycles using miRNA-specific primers and Taqman PCR master mix, in a 7300Real Time PCR System (Applied Biosystems). PCR reactions for each sample were performed in triplicate and expression values were normalized to RNU6B.

### Development of an *in vitro* MRCC sunitinib-resistant cell model and analysis of miRNA expression

Caki-2 cells isolated from MRCC cell lines (ATCC, Manassas, VA, USA) were cultured in McCoy's 5a Medium modified with L-Glutamine (Biowhitaker) supplemented with 10% Fetalclone III (Hyclone) and 1% of Penicillin-Streptomycin (Gibco). A sunitinib-resistant Caki-2 cell line (SRCaki-2) was generated by continuous exposure for four months to gradually increasing doses of sunitinib: From 8 µM to 14 µM (Sutent, LC laboratories). The initial dose of 8 µM, corresponding to the LD_50_, was selected from a previous MTT assay, where sunitinib cytotoxicity was tested with doses ranging from 0.001 µM to 20 µM for 72 hours.

Total RNA from Caki-2 and SRCaki-2 cells was obtained by the Trizol extraction method (Invitrogen) and expression of the selected miRNAs was studied by qRT-PCR. Expression results were normalized to RNU6B levels.

### miR-942 overexpression in MRCC Caki-2 cell line

Pre-miR-942 and pre-miR Negative Control (5 nmol, Ambion) were transfected into parental Caki-2 cells grown in six-well plates (seeded 24 hours before at 2×10^5^ cells/well) with Lipofectamine 2000 (Invitrogen). Total RNA was extracted after 72 hours and transfection efficiency was verified by qRT-PCR. Sunitinib sensitivity of transfected cells was studied by MTT assay following doses and protocol described before.

### Gene expression by qRT-PCR

Expression of sunitinib-targeted tyrosine kinase receptors, Platelet-derived Growth Factor Receptors (PDGFRα and β), Vascular Endothelial Growth Factor Receptors (VEGF-R 1, 2, 3), c-Kit, FLT3 and levels of the cytokines matrix metalloproteinase-9 (MMP-9), tumour necrosis factor-α (TNF-α) and interleukin-8 (IL-8), were measured by qRT-PCR.

### Western blot and zymogram

After 48 h incubation, serum-free cell supernatants and extracts from transfected cells were recovered to perform subsequent VEGF and MMP-9 western blot analysis (S1 Text). Gelatin zymography (Invitrogen) of secreted proteins was analysed to check MMP-9 gelatinolytic activity (S2 Text).

### Co-culture and cell migration assays

HBMEC endothelial cells kindly donated by Dr. Lecanda (Laboratory of Cell Adhesion and Metastasis, CIMA) were grown on 0.2% gelatine-coated plates in EBM-2 medium supplemented with 5%FBS. MiR-942/Caki-2 cells and HBMEC were co-cultured in 0.4 µm pore size 6-well Boyden chambers. HBMEC (1.5×10^5^ cell/well) were plated at the bottom of the plates in EBM-2 medium. After 2 h, 8×10^4^ miR-942-overexpressing Caki-2 cells or control cells (mock-transfected) were seeded in McCoy's 5a Medium in the inserts. Co-culture was incubated for 72 hours and HBMEC cells were then harvested to perform MTT (with sunitinib doses up to 35 µM), western blot for p-VEGFR2, total VEGFR2, p-p44/p42 MAPK (Erk1/2) and total Erk1/2 (S3 Text), and for migration assays.

Migration was performed in 24 well Boyden chambers. HBMEC (5×10^4^ cells) were seeded in the chamber in a final volume of 150 µl in serum free medium. The lower compartment was filled with 450 µl of 5% serum containing EBM-2 medium to chemoattract the cells. 48 h later, cells from the insert were removed with a swab and cells that migrated to the lower compartment were fixed with 4% formalin, stained with crystal violet and counted with a Leica DMIL LED microscopy.

### Statistical methods

Statistical differences in normalized miRNA expression between extreme phenoytpes were examined with the Mann-Whitney *U-*test for unpaired non-parametric variables and the Student's *t*-test for unpaired data for parametric variables. Normality was tested with the Shapiro-Wilks Test. Data were analysed with SPSS software, version 15.0. P-values<0.05 were considered statistically significant. Survival variables were studied with the Kaplan-Meier test and curves were compared with the Log-rank test. The study was designed and the results reported following REMARK guidelines.

TLDAs data were analysed with R/Bioconductor [17], performing a filtering process to eliminate the miRNAs not detected in the experiment, followed by normalization and LIMMA statistical analysis [18]. A p-value threshold of 0.05 was established to select differentially expressed miRNAs.

A machine learning algorithm based on logistic regression was applied to classify patients and to identify the optimal separating miRNAs between extreme phenotypes. The performance of the classifiers was evaluated using Receiver Operator Characteristics Curve (ROC) analysis.

## Results

### Screening of miRNAs with TLDAs

TLDA profiling of 673 miRNA in tumors from six patients belonging to the screening cohort presenting extreme phenotypes of marked sunitinib efficacy (n = 3: 7.3%) or resistance (n = 3: 7.3%) identified 64 miRNAs differentially expressed (p<0.05) between both groups (data deposited in NCBI's Gene Expression Omnibus and accessible through GEO Series accession number GSE37766). Seven candidates (miR-133a, miR-628-5p, miR-942, miR-484, miR-146a-5p, miR-374a and miR-486-5p) were selected for further study based on these criteria: Differential expression obtained from the TLDA analysis; bioinformatic-based target prediction databases combined with bibliographic search to identify microRNAs related to MRCC development or sunitinib-targeted pathways.

### Assessment of miRNAs by qRT-PCR in the independent series

**Figure 1:**
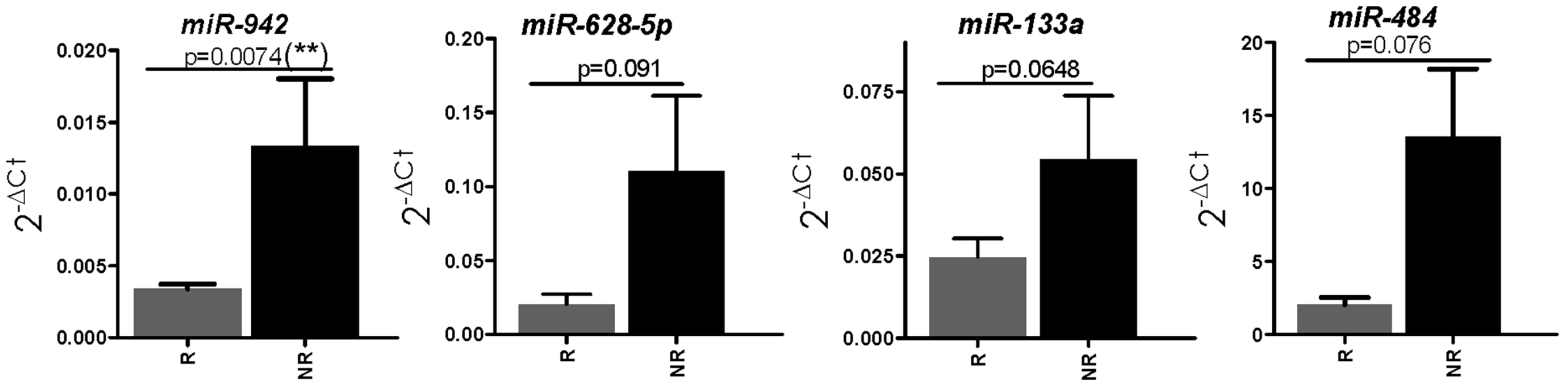
Tumor microRNA expression in MRCC patients with extreme phenotypes treated with sunitinib from the independent cohort. , (sunitinib sensitive, n = 14 and sunitinib resistant, n = 6). MiR-942, miR-628-5p, miR-133a and miR-484 were overexpressed in sunitinib resistant patients. MiR-942 significantly discriminated between both patients groups. MiRNA levels are represented as 2^−ΔCt^ mean ± SEM. MiRNA expression was normalized against RNU6B. R = responders. NR = non responders.

Selected miRNAs were studied by qRT-PCR in tumors from patients with extreme phenotypes of sunitinib response (n = 14, 11.9%) and resistance (n = 6, 5.1%) belonging to the independent series. Increased levels of miR-942, miR-133a, miR-628-5p and miR-484 were observed in resistant phenotypes as compared with sensitive phenotypes (Figure 1), achieving statistical significance for miR-942 (p = 0.0074). The remaining miRNAs showed borderline significant p-values (Figure 1). The tendency of the expression of some miRNAs was opposite to that found in the TLDAs. No differences were found in the other miRNAs (Figure S1A). Prediction of efficacy was superior for miR-942 alone [Area Under the ROC curve (AUROCC = 0.798, sensitivity (S) = 92%, specificity (Sp) = 50%] than for the 4-miRNA combination (AUROCC = 0.619, S = 85%, Sp = 50%).

**Figure 2:**
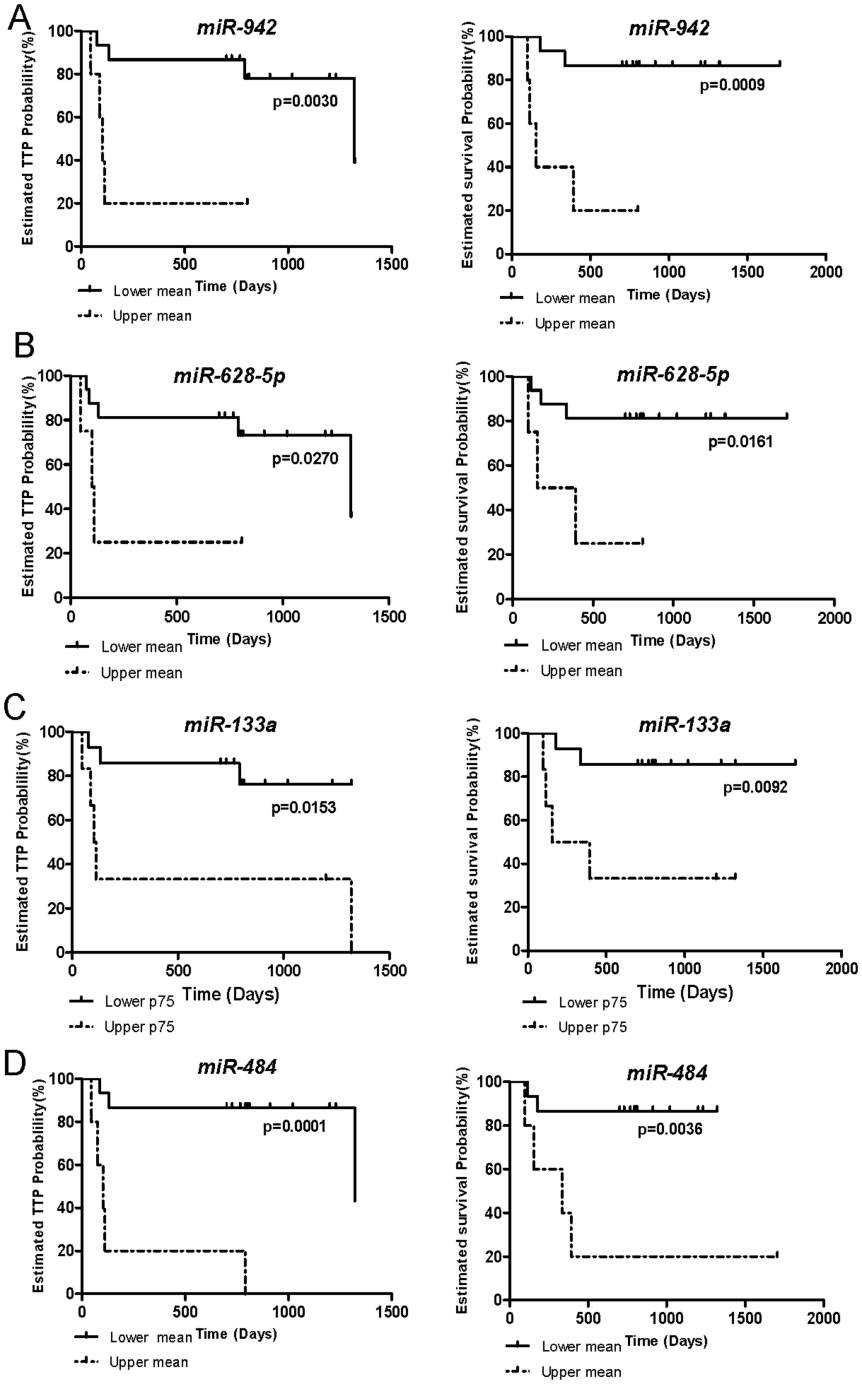
Association between miRNA expression and TTP and OS. TTP and OS were significantly reduced in MRCC patients treated with sunitinib that had (**A**) miR-942 expression over the mean; (**B**) miR-628-5p levels over the mean; (**C**) miR-133a levels over p75; (**D**) and miR-484 expression over the mean.

High expression of miR-133a, miR-628-5p, miR-942 and miR-484 was significantly associated with reduced TTP and OS (Figure 2 and table S1). No correlation was found between expression of the other miRNAs and survival variables (data not shown).

### miRNAs expression in sunitinib-resistant Caki-2 cells

We established a sunitinib-resistant Caki-2 cell line (SRCaki-2) to validate our results. 90% of SRCaki-2 cells were alive after 72 hours of sunitinib exposure at 12 µM dose, as compared with only 12% of cells in the sensitive parental Caki-2 (Figure S2).

**Figure 3:**
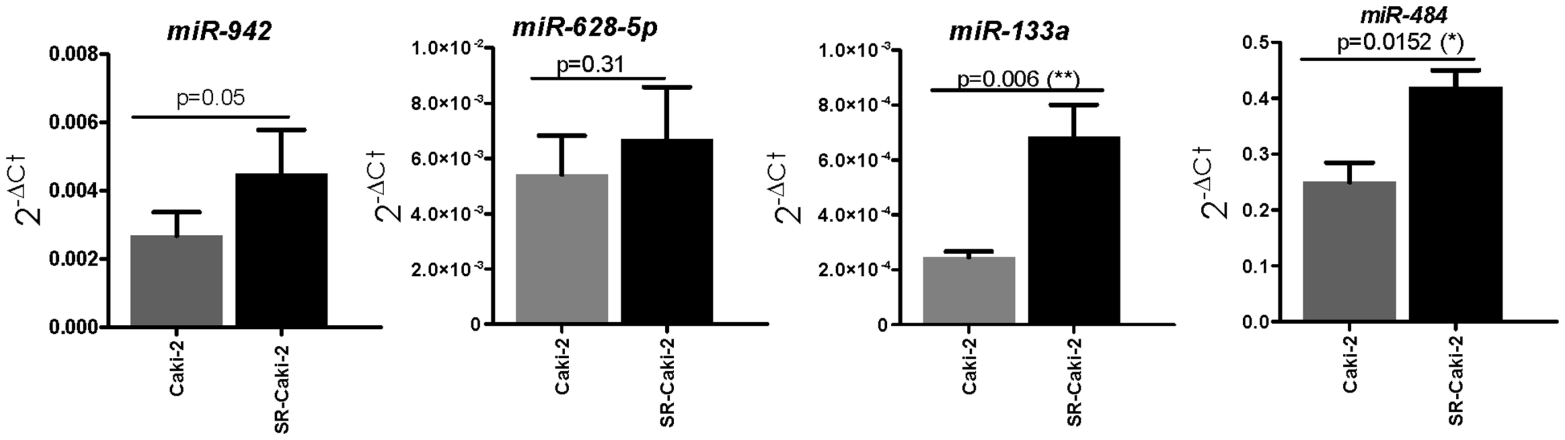
MicroRNA levels in the sunitinib-resistant Caki-2 cell line (SRCaki-2) and its sunitinib-sensitive counterpart. MiR-942, miR-133a and miR-484 were significantly overexpressed in resistant cells. MiRNAs expression is showed as 2^−ΔCt^ mean ± SEM. Expression was normalized against RNU6B.

We quantified levels of the 7 selected miRNAs by qRT-PCR in sensitive and resistant cells. Overexpression of miR-133a (p = 0.006), miR-484 (p = 0.0152) and miR-942 (p = 0.05) and downregulation of miR-486-5p (p = 0.017) was found in resistant cells in comparison with sensitive cells (Figure 3 and S1B), as was the case for patients showing resistance. No statistically significant differences were observed for the other three miRNAs, although the expression trend was similar to that observed in patients (Figure S1B).

### miR-942 overexpression in Caki-2 cells promotes MMP-9 and VEGF secretion

**Figure 4:**
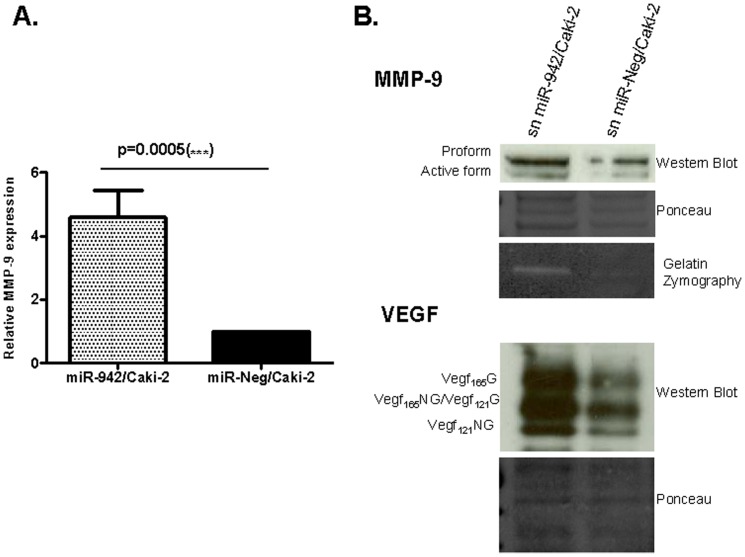
MMP-9 increases in miR-942/Caki-2 cell line. (**A**) MMP-9 mRNA levels in miR-942/Caki-2 cells were significantly increased in comparison to miR-Neg/Caki-2. Data are represented as 2^−(ΔΔCt)^ using GAPDH as housekeeping. (**B**) Secreted MMP-9 (both the 92 KDa proform and the active form 86 KDa) and VEGF isoforms (VEGF_165_G/23 KDa, VEGF_165_NG/VEGF_121_G/18 KDa, VEGF_121_NG/15 KDa) levels are higher in miR-942/Caki-2 than in the negative control. Proteolytic activity of secreted MMP-9 was proven by gelatin-zymography.

We then studied the mechanism by which miR-942 could contribute to sunitinib resistance. With this aim, pre-miR-942 was transfected to Caki-2 cells to induce miR-942 overexpression. MTT assays showed that the increase of miR-942 did not modify sunitinib cytotoxicity on Caki-2-transfected cells (Figure S2). We next evaluated the expression of PDGFR (a predicted target for this microRNA), but found no expression in these cells. Moreover, no significant expression of the other sunitinib-targeted receptors was observed (results not shown). These findings suggested that resistance to sunitinib mediated by miR-942 upregulation might be related to a paracrine effect. To try to identify alternative pathways regulated by miR-942, we studied the expression of pro-angiogenic cytokines that have been previously described to confer resistance to sunitinib in cancer patients, including MMP-9, TNF-α [10] and IL-8 [19]. mRNA levels of MMP-9 were significantly increased (p = 0.0005) in miR-942-overexpressing cells compared to controls (Figure 4A), whereas no differences were observed for TNF-α and IL-8 (data not shown).

Confirmation of this result by Western blot is shown in Figure 4B: miR-942/Caki-2 cells secreted more MMP-9 as well as its downstream regulated gene VEGF than miR-Neg/Caki-2 cells. Supernatants from miR-942/Caki-2 cells were enriched in both pro-MMP-9 (92 KDa) and activated-MMP-9 (86 KDa) and glycosylated/non-glycosylated VEGF_121_ (15 KDa) and VEGF_165_ (23 KDa), as well as the non-glycosilated VEGF_165_/glycosilated VEGF_121_ (18 KDa). In addition, proteinase activity of soluble MMP-9 evaluated by gelatin-zymography demonstrated a stronger band (86 KDa) for miR-942/Caki-2 cells than that of miR-Neg/Caki-2 cells (Figure 4B). All these results show that miR-942 induces secretion of both active MMP-9 and VEGF.

To assess whether this regulatory system would also be functional in the Caki-2 resistant cells that we generated, expression of MMP-9 and VEGF in SRCaki-2 was checked. Levels of miR-942 were slightly increased (1.5 fold) in these cells, and levels of MMP-9 and VEGF were similar to parental control (data not shown). This result suggests that SRCaki-2 cells, which were generated by continuous exposure to the drug, may have acquired resistance through mechanisms different to miR-942 upregulation.

### Paracrine regulation of miR-942-mediated MMP-9 and VEGF secretion by tumor cells on endothelial cells

**Figure 5:**
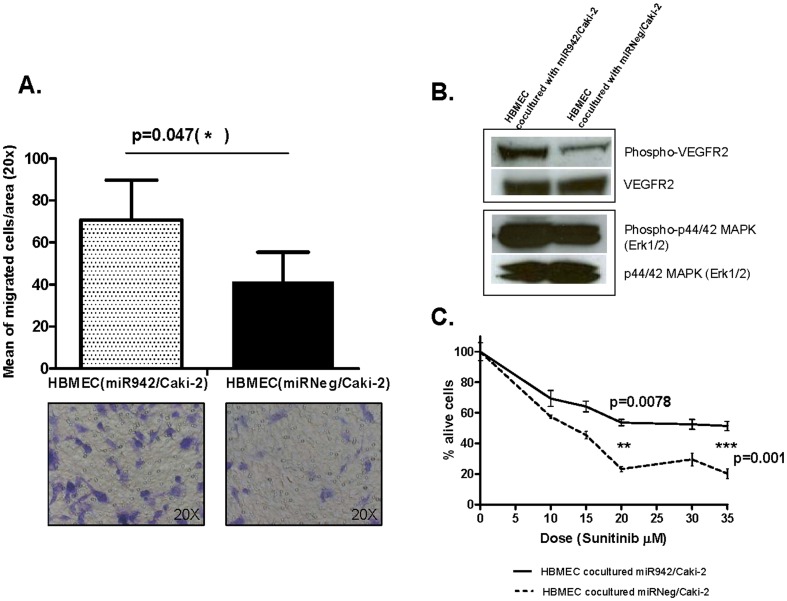
Functional effects of secreted MMP-9 and VEGF on endothelial cells. (**A**) Migration of HBMEC was significantly increased after co-culture with miR-942/Caki-2 (*: p<0.05), as compared with co-culture with miR-Neg/Caki-2 cells. (**B**) HBMEC became less sensitive to sunitinib activity after co-culture with miR-942/Caki-2 (LD_50_>35 µM) in comparison to HBMEC co-cultured with miR-Neg/Caki-2 cells (LD_50_ 13.5 µM). (**C**) Phosphorylation of VEGFR2 and p44/42 MAPK (Erk1/2) proteins was increased in HBMEC co-cultured with miR-942/Caki-2, as compared to the co-culture with miR-Neg/Caki-2 cells.

We next investigated whether high levels of secreted active MMP-9 and VEGF by miR-942/Caki-2 cells would have functional effects on endothelial cells. HBMEC migration ability was significantly increased (p = 0.047) 72 hours after co-culture with miR-942/Caki-2 (Figure 5A) in comparison to miR-Neg/Caki-2 cells. Increased levels of phosphorylated VEGFR2 (230 KDa) and phosphorylated p44/42 MAPK (Erk 1/2) (42–44 KDa) was observed in HBMEC after co-culture with miR-942/Caki-2 (Figure 5B). These findings indicate that miR-942 overexpression by MRCC cells promotes migration of endothelial cells through MMP-9 and VEGF overexpression.

**Figure 6:**
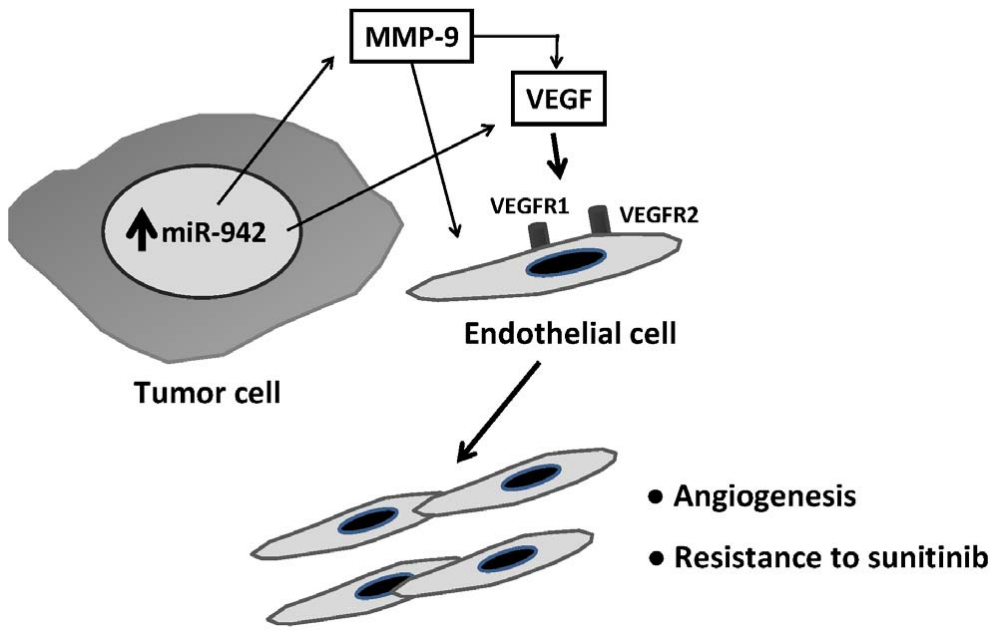
Proposed mechanism for the miR-942-mediated MMP-9/VEGF paracrine regulation of cancer cells and endothelial cells.

Moreover, HBMEC co-cultured with miR-942/Caki-2 cells were less sensitive to the cytotoxic effect of sunitinib (LD_50_>35 µM) than those co-cultured with miR-Neg/Caki-2 cells (LD_50_ 13.5 µM) (Figure 5C). Based on our results, we propose a model that could explain how MRCC tumor cells with high miR-942 expression may act through paracrine mechanisms to enhance angiogenesis and resistance to sunitinib (Figure 6).

## Discussion

MiRNAs play a relevant role in the pathogenesis and the natural history of MRCC. Differential expression of miRNAs in MRCC compared to normal kidney has been described by previous studies [20] and miRNA signatures can accurately classify different histological subtypes of MRCC [21], [22]. Moreover, miRNAs have been related to the risk of developing early metastasis following nephrectomy [23] and with OS and TTP [24], [25]. However, there is very limited information about the role of miRNAs as predictive biomarkers of treatment efficacy in MRCC.

miRNAs posses both advantages and disadvantages as potential biomarkers. Minute amounts of paraffin-embedded tissue can be used to measure a large number of miRNAs, but the lack of standardization in methods and cut-off values may hinder their potential as biomarkers. Despite these potential problems, an increasing number of studies are exploring microRNAs as predictive biomarkers in different cancer types. miR-26 was reported to predict interferon-α efficacy in hepatocellular carcinoma [26]. High tumor levels of miR-92a-2* are associated with chemoresistance and decreased survival in NSCLC and the miR-200 family might constitute a predictive biomarker of paclitaxel-based therapy in ovarian carcinoma [27]. Gamez-Pozo et al. have recently described that expression of particular miRNA signatures in peripheral blood predicts sunitinib activity in MRCC [2].

We have identified candidate tumor miRNAs predictive of sunitinib efficacy in MRCC through screening of 673 miRNAs using TLDAs. We selected MRCC patients with extreme phenotypes of marked efficacy or primary resistance to sunitinib. By focusing on these patient subsets, we increased the probability of finding clinically relevant results and reduced the number of screened patients and the costs of using expensive high-throughput platforms. The rationale behind the use of a high-throughput technique is to increase the chance of finding relevant predictive factors by exploring a large number of potential candidates. We found 64 miRNAs differentially expressed between both groups and seven of these were selected for further analysis based on results obtained from bioinformatic-based target prediction databases and by their relationship with MRCC development and sunitinib-targeted pathways.

qRT-PCR in an independent cohort showed that miR-942, miR-133a, miR-628-5p, and miR-484 levels were increased in tumors from sunitinib resistant patients. High expression of these miRNAs was significantly associated with OS and TTP. ROC analysis showed that miR-942 alone could accurately classify the selected extreme phenotypes and thus, warrants further study as a predictive biomarker of sunitinib activity. Interestingly, our findings in patients were replicated in a sunitinib-resistant Caki-2 cell line developed for this purpose.

Bioinformatic databases predict that, among other genes, miR-942 targets PDGFR. However, both MRCC Caki-2 and endothelial HBMEC [28] cells lack PDGFR; moreover, all other sunitinib-targeted receptors were not expressed (or were almost undetectable) in Caki-2 cells as well. Therefore, the mechanisms of resistance cannot be related in this experimental system with a miR-942-mediated decrease in PDGFR levels, which would lead to sunitinib resistance. In addition, we found that sensitivity of Caki-2 cells to sunitinib was not modified by miR-942 overexpression.

In search for a possible alternative mechanism, we quantified levels of angiogenic factors that have been previously reported to be associated with sunitinib in patients, such as MMP-9, TNF-α and IL-8. Overexpression of miR-942 in Caki-2 cells caused a significant upregulation of MMP-9 (and its downstream angiogenic factor VEGF) in these cells. Co-culture experiments using Caki-2 and HBMEC cells demonstrated the existence of a paracrine loop activated by miR-942 that resulted in activation of VEGFR-mediated signalling cascades, endothelial cell migration and resistance to sunitinib. It is known that MMP-9 is highly produced and secreted by malignant cells, and that its overexpression correlates with poor prognosis and survival in several cancer types [29]. This metalloproteinase regulates VEGF bioavailability and release, leading to angiogenesis and metastasis [30]. Jadhav et al showed that inhibition of MMP-9 results in a reduction of *in vitro* endothelial cell invasion and tube formation, thus demonstrating that MMP-9 plays a key role in endothelial cell networking and angiogenesis [31].

We then provide in the present study first evidence of a novel paracrine mechanism that involves the activation of miR-942/MMP-9/VEGF axis in cancer cells to promote endothelial cell migration and resistance to sunitinib. In line with our findings *in vitro*, we demonstrated in a previous study that high circulating MMP-9 levels are significantly associated with lack of response to sunitinib and shorter TTP and OS in MRCC patients [10]. Taken together, these data strongly suggest that MMP-9 can be a key factor for sunitinib resistance.

The question about how increased levels of miR-942 lead to MMP-9 and VEGF upregulation still remains to be solved. Our preliminary results (unpublished observations) using microarray analysis combined with identification of potential miR-942 targets suggest a downregulation of several genes of the Fanconi Anemia/BRCA pathway [32]. Transcriptional repression of this pathway has been shown to induce MMP-9/VEGF levels [33], [34], which may offer a possible mechanistic explanation about miR-942 effects. However, this unexplored possibility needs to be addressed in future work.

Our study has some limitations. Apart from the lack of standardization (as indicated), the strategy of extreme phenotype selection, while potentially useful, also requires further development, e.g: precise definition of what constitutes an extreme phenotype, comparability between different studies, optimal calculation of sample sizes for the screening and validation cohorts, and extrapolation of the findings in extreme phenotypes to the general population. Nevertheless, our strategy has successfully led us to the identification of novel potential predictive factors of sunitinib activity.

In conclusion, we have identified a set of four tumor miRNAs with predictive potential of sunitinib efficacy in MRCC, from which miR-942 is the most promising candidate. We have also demonstrated that high levels of miR-942 in tumor MRCC cells upregulates both MMP-9 and VEGF to induce endothelial cell migration and resistance to sunitinib. This study also confirms that extreme phenotype selection in combination with high-throughput analysis is a valid strategy to identify candidate predictive biomarkers in cancer research.

## Supporting Information

Text S1 **Western Blot analysis for MMP-9 and VEGF isoforms from pre-miR-942 and pre-miR-mock transfected Caki-2 cells.**
(DOCX)

Text S2 **Gelatin zymography to evaluate MMP-9 activity from pre-miR-942 and pre-mir-mock-transfected Caki-2 cells (negative control).**
(DOCX)

Text S3 **Western Blot analysis for phospho-VEGFR2, total VEGFR2, phospho-p44/42 MAPK (Erk1/2) and total Erk1/2 from HBMEC cells co-cultured with miR-942/Caki-2 and miR-Neg/Caki-2 cell lines.**
(DOCX)

Figure S1 (**A**) MiR-146a-5p, miR-374a and miR-486-5p tumor expression in MRCC sunitinib sensitive and resistant patients. No differences were found in the expression of any of these miRNAs between both groups. Expression is represented as 2^−ΔCt^ (mean ± SEM). MiRs levels were normalized against RNU6B. (**B**) Mir-146a-5p, miR-374a and miR-486-5p levels in parental and resistant Caki-2 cells. Mir-486-5p was significantly downregulated in resistant cells compared to the sunitinib sensitive parental cell line. MiR-146a-5p and miR-374a expression was not different between both cell lines. Data are shown as 2^−ΔCt^ mean ± SEM. MiRNA levels were normalized to RNU6B. R = responders; NR = non responders.
(TIF)

Figure S2 **MTT citotoxicity assay in Caki-2, SRCaki-2, miR-942/Caki-2 and miR-Neg/Caki-2 cells (doses up to 20 µM), analyzed 72 h after administration of the drug.** MiR-942 overexpression in Caki-2 did not increases resistance to sunitinib.
(TIF)

Table S1 **Median time to progression and overall survival according to expression of miR-942, miR-133a, miR-628-5p and miR-484.**
(DOC)
