# Supplementary material for: Identification of Tissue microRNAs Predictive of Sunitinib Activity in Patients with Metastatic Renal Cell Carcinoma
Source: PLoS One. 2014 Jan 24;9(1):e86263. doi: 10.1371/journal.pone.0086263 (PMC3901669; doi:10.1371/journal.pone.0086263)
Supplement: Text S1 — Western Blot analysis for MMP-9 and VEGF isoforms from pre-miR-942 and pre-miR-mock transfected Caki-2 cells. (DOCX) [file pone.0086263.s001.docx]

**S1 Text.**

**Western Blot analysis for MMP-9 and VEGF isoforms from pre-miR-942 and pre-miR-mock transfected Caki-2 cells.** Transfected Caki-2 cells were incubated in serum free culture media for 48 hours. Cell supernatants were collected and concentrated by several centrifugations for 10m at 3000g and 4ºC, through Amicon Ultra 10K tubes (Millipore) to a final volume of 400µl.

Cells from the same 6-well plates were harvested and lysed in lysis buffer (RIPA buffer 100ml; Tris 1M, 0.8775g NaCl, 1ml Triton X-100, 1g Sodium deoxycholate, 0.1g SDS, 1mM phenylmethasulfonylfluoride, 25mM NaF, distilled water) and treated with protease and phosphatase inhibitors (Sigma) for 30min at 4°C. Thirty micrograms of proteins from supernatants and cell pellets were separated through 12% acrylamide gel, transferred to a membrane and incubated with rabbit polyclonal antibodies to detect MMP-9 (1:1000, Neomarkers) and VEGF (1:500 Santa Cruz), followed by HRP-conjugated secondary antibodies at 1:3000 dilution. The signal was visualised by enhance chemiluminescence using LumiLight plus Kit (Roche).
