# Supplementary material for: Identification of Tissue microRNAs Predictive of Sunitinib Activity in Patients with Metastatic Renal Cell Carcinoma
Source: PLoS One. 2014 Jan 24;9(1):e86263. doi: 10.1371/journal.pone.0086263 (PMC3901669; doi:10.1371/journal.pone.0086263)
Supplement: Text S2 — Gelatin zymography to evaluate MMP-9 activity from pre-miR-942 and pre-mir-mock-transfected Caki-2 cells (negative control). (DOCX) [file pone.0086263.s002.docx]

**S2 Text.**

**Gelatin zymography to evaluate MMP-9 activity from pre-miR-942 and pre-mir-mock-transfected Caki-2 cells (negative control).** Fifteen micrograms of protein from concentrated serum free medium obtained after pre-miR-942 transfection of Caki-2 cells were subjected to SDS-PAGE using a 10% zymogram gelatin gel (Novex, Invitrogen). After electrophoresis, the gel was soaked in 50 mM Tris–HCl (pH 7.5) containing 2.5% Triton X-100 at room temperature with gentle shaking for 30m, and then incubated overnight in 50 mM Tris–HCl (pH 7.5) containing 5 mM CaCl_2_ and 0.2 M NaCI at 37°C. The gels were then stained with Coomassie Brilliant Blue.
