# Supplementary material for: Identification of Tissue microRNAs Predictive of Sunitinib Activity in Patients with Metastatic Renal Cell Carcinoma
Source: PLoS One. 2014 Jan 24;9(1):e86263. doi: 10.1371/journal.pone.0086263 (PMC3901669; doi:10.1371/journal.pone.0086263)
Supplement: Text S3 — Western Blot analysis for phospho-VEGFR2, total VEGFR2, phospho-p44/42 MAPK (Erk1/2) and total Erk1/2 from HBMEC cells co-cultured with miR-942/Caki-2 and miR-Neg/Caki-2 cell lines. (DOCX) [file pone.0086263.s003.docx]

**S3 Text.**

**Western Blot analysis for phospho-VEGFR2, total VEGFR2, phospho-p44/42 MAPK (Erk1/2) and total Erk1/2 from HBMEC cells co-cultured with miR-942/Caki-2 and miR-Neg/Caki-2 cell lines.**

Forty micrograms of total proteins from HBMEC were obtained after 72h of co-culture with miR-942/Caki-2 or miR-Neg/Caki-2 cells. Samples were separated through a 12% acrylamide gel following same protocol explained in S1 Text. Rabbit polyclonal antibodies to detect phospho-VEGFR2, VEGFR2 (both at 1:500, Cell Signaling) and phospho-p44/42 MAPK (Erk1/2), Erk1/2 (both at 1:2000, Cell Signaling) were used, followed by HRP-conjugated secondary antibodies at 1:2000 dilution.
