# Supplementary figures and images for: Identification of Tissue microRNAs Predictive of Sunitinib Activity in Patients with Metastatic Renal Cell Carcinoma
Source: PLoS One. 2014 Jan 24;9(1):e86263. doi: 10.1371/journal.pone.0086263 (PMC3901669; doi:10.1371/journal.pone.0086263)

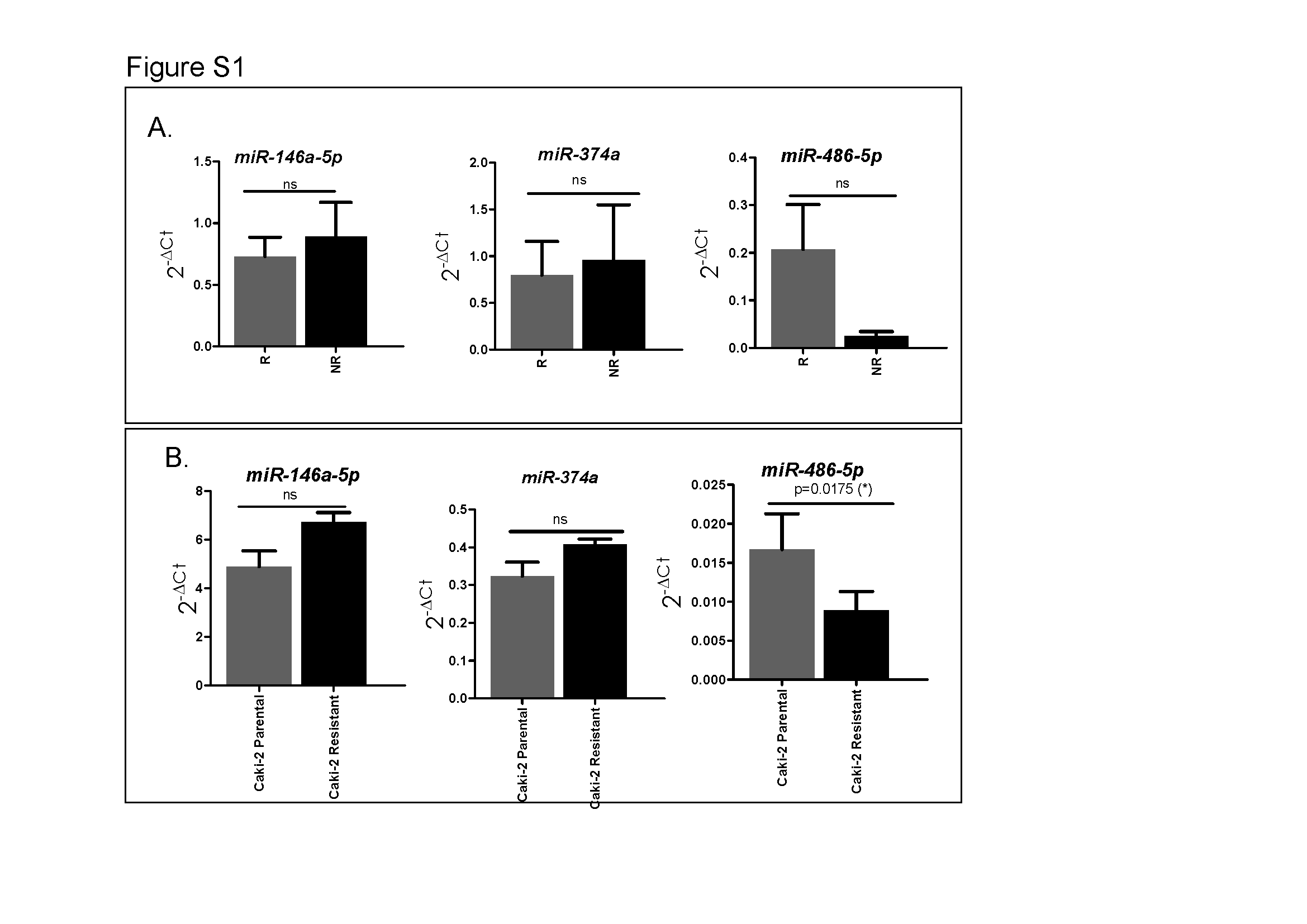

Supplement: Figure S1 — (A) MiR-146a-5p, miR-374a and miR-486-5p tumor expression in MRCC sunitinib sensitive and resistant patients. No differences were found in the expression of any of these miRNAs between both groups. Expression is represented as 2−ΔCt (mean ± SEM). MiRs levels were normalized against RNU6B. (B) Mir-146a-5p, miR-374a and miR-486-5p levels in parental and resistant Caki-2 cells. Mir-486-5p was significantly downregulated in resistant cells compared to the sunitinib sensitive parental cell line. MiR-146a-5p and miR-374a expression was not different between both cell lines. Data are shown as 2−ΔCt mean ± SEM. MiRNA levels were normalized to RNU6B. R = responders; NR = non responders. (TIF) [file pone.0086263.s004.tif]
