# Supplementary material for: Identification of Tissue microRNAs Predictive of Sunitinib Activity in Patients with Metastatic Renal Cell Carcinoma
Source: PLoS One. 2014 Jan 24;9(1):e86263. doi: 10.1371/journal.pone.0086263 (PMC3901669; doi:10.1371/journal.pone.0086263)
Supplement: Table S1 — Median time to progression and overall survival according to expression of miR-942, miR-133a, miR-628-5p and miR-484. (DOC) [file pone.0086263.s006.doc]

**Supplementary Table 1.** Median time to progression and overall survival according to expression of miR-942, miR-133a, miR-628-5p and miR-484.
